# Detection of a Putative TetR-Like Gene Related to *Mycobacterium bovis* BCG Growth in Cholesterol Using a *gfp*-Transposon Mutagenesis System

**DOI:** 10.3389/fmicb.2017.00315

**Published:** 2017-03-06

**Authors:** Isabel Otal, Esther Pérez-Herrán, Lazaro Garcia-Morales, María C. Menéndez, Jorge A. Gonzalez-y-Merchand, Carlos Martín, María J. García

**Affiliations:** ^1^Grupo de Genética de Micobacterias, Departamento de Microbiologia, Medicina Preventiva y Salud Pública, Universidad de ZaragozaZaragoza, Spain; ^2^Centros de Investigación Biomédica en Red Enfermedades Respiratorias, Instituto de Salud Carlos IIIMadrid, Spain; ^3^Instituto de Investigación Sanitaria AragónZaragoza, Spain; ^4^Diseases of the Developing World, GlaxoSmithKlineTres Cantos, Spain; ^5^Departamento de Microbiología, Escuela Nacional de Ciencias Biológicas, Instituto Politécnico NacionalCiudad de Mexico, Mexico; ^6^Departamento de Medicina Preventiva, Universidad AutónomaMadrid, Spain

**Keywords:** transposon mutagenesis, *gfp*, *Mycobacterium bovis* BCG, cholesterol, BCG_2177c gene, TetR-family

## Abstract

*In vitro* transposition is a powerful genetic tool for identifying mycobacterial virulence genes and studying virulence factors in relation to the host. Transposon shuttle mutagenesis is a method for constructing stable insertions in the genome of different microorganisms including mycobacteria. Using an IS*1096* derivative, we have constructed the Tn*gfp*, a transposon containing a promoterless green fluorescent protein (*gfp*) gene. This transposon was able to transpose randomly in *Mycobacterium bovis* BCG. Bacteria with a single copy of the *gfp* gene per chromosome from an *M. bovis* BCG::Tngfp library were analyzed and cells exhibiting high levels of fluorescence were detected by flow cytometry. Application of this approach allowed for the selection of a mutant, BCG_2177c::Tn*gfp* (BCG-Tn), on the basis of high level of long-standing fluorescence at stationary phase. This BCG-Tn mutant showed some particular phenotypic features compared to the wild type strain, mainly during stationary phase, when cholesterol was used as a sole carbon source, thus supporting the relationships of the targeted gene with the regulation of cholesterol metabolism in this bacteria. This approach showed that Tn*gfp* is a potentially useful tool for studying the involvement of the targeted loci in metabolic pathways of mycobacteria.

## Introduction

Tuberculosis (TB) remains a leading cause of morbidity and mortality among communicable diseases, and it is currently ranked the major killer among worldwide infectious diseases caused by a single pathogen^1^. The HIV/AIDS pandemic, the deterioration of public health systems in developing countries and the emergence of multi (MDR), extensively (XDR), and totally (TDR) drug–resistance strains have further contributed to the spread of the pathogen and reinforced the urgent need for more efficient vaccines and treatment against TB (Gunther, 2014). Moreover, latently infected people represent a dangerous reservoir of TB (Garcia and Lopez, 2016). The human population is estimated at approximately 7 billion by the United Nations, with over one third being infected with the tubercle bacilli. Therefore, it can be assumed that more than 2 billion people have latent TB infection (LTBI). Based on this statistics it is not unusual that understanding and controlling LTBI were considered as core parts of the End TB Strategy, launched from 2016 to 2035 by WHO (Garcia and Lopez, 2016).

Cholesterol has been identified as an essential carbon source for LTBI in which the bacteria exist in a non-replicative state termed dormancy (Nesbitt et al., 2010). Several studies have demonstrated that *Mycobacterium tuberculosis* mutants lacking the capacity to acquire or degrade cholesterol are defective for growth in some animal models of TB (Pandey and Sassetti, 2008; Chang et al., 2009; Nesbitt et al., 2010). Furthermore it has been shown that the metabolism of host-derived carbon sources such as fatty acids and/or cholesterol is crucial for *M. tuberculosis* persistence in macrophages during chronic infections (Schnappinger et al., 2003; Rengarajan et al., 2005; Pandey and Sassetti, 2008; Miner et al., 2009; Griffin et al., 2012; Rohde et al., 2012).

The *M. tuberculosis* complex (MTBC) is characterized by 99,95% sequence similarity at the nucleotide level and a strictly clonal population structure (Brosch et al., 2002). Continuous advances in developing biological tools and reagents have facilitated genetic manipulation of the MTBC (Bardarov et al., 1997; Pelicic et al., 1997). These advances, in combination with the availability of whole genome sequences of multiple strains of members of the MTBC^2^ have allowed for the study of the contribution of individual genes to the *M. tuberculosis* pathogenicity, including those related to dormancy, the status of the bacilli during LTBI (Forrellad et al., 2013). One of the *in vitro* conditions required to mimic the dormant state is the stationary phase of growth in the presence of a lipid environment (Rodriguez et al., 2014).

Transposon mutagenesis is a classical procedure to study gene-function of bacteria under a global perspective (Lin et al., 2014; Kwon et al., 2016). In addition, genetic fusions have provided an important means of analyzing basic biological systems from the earliest times of molecular genetics. The basic principle of the genetic fusion approach was to put an assayable gene product under the control of another gene or promoter of interest (named reporter) thus supplying a means to monitor its expression. In this work, we have studied the potential mutagenicity supplied by a transposon that includes a promoter-less *gfp* gene (“*g*reen *f*luorescent *p*rotein”) as a reporter gene.

The GFP from the jellyfish *Aequorea victoria* has been extensively used as a useful marker in studies of various biological processes (Cubitt et al., 1995; Valdivia and Falkow, 1998). GFP is a 238-amino-acid-long protein encoded by the *gfp* gene (Prasher et al., 1992). When excited by blue light at a wavelength of 395 nm, the protein emits green light that can be observed *in vivo* since no substrates or cofactors are needed for the unique post-translational modification that generates the fluorescent chromophore (Cody et al., 1993). GFP has been successfully expressed in a wide range of organisms such as the nematode *Caenorhabditis elegans* (Chalfie et al., 1994), the small fly *Drosophila* (Wang and Hazelrigg, 1994; Bach et al., 2007), and pathogenic mycobacteria (Dokladda et al., 2015). Modified transposons containing truncated reporter genes could be useful to identify promoters and study gene expression in mycobacteria. The transposons Tn*5367* and Tn*5368* contain IS*1096* (Cirillo et al., 1991) and both showed to be active in *M. bovis* BCG generating random insertions and giving rise to a set of auxotrophic mutants (Mcadam et al., 1995). These transposons have also been active in several strains of *M. tuberculosis* (Bardarov et al., 1997; Pelicic et al., 1997).

In the present work, we describe the construction of an IS*1096*-derivative transposon harboring the reporter *gfp* gene and its delivering system using a thermo/sucrose sensitive mycobacterial plasmid. This transposon was checked and analyzed in the slow-growing non-pathogen *M. bovis* BCG, belonging to MTBC, which can be manipulated and results can be extrapolated to *M*. *tuberculosis*. Using flow cytometry, we were able to differentiate populations of fluorescent mycobacteria. Applying this procedure, we isolated the *M. bovis* BCG_2177c::Tn*gfp* (BCG-Tn) a mutant with high fluorescence at stationary growth phase. The targeted gene, BCG_2177c, a homologous of Rv2160A of *M. tuberculosis*, has been previously identified as a putative member of the TetR-family of transcriptional regulators (Balhana et al., 2015) and members of this family have been associated with the regulation of genes involved in cholesterol metabolism (Casabon et al., 2013; Wipperman et al., 2014). Furthermore, phenotypic and genotypic characterization of the mutant in this work supports the involvement of this gene in the metabolism of cholesterol in *M. bovis* BCG.

## Materials and Methods

### Bacterial Strains, Plasmids, and Growth Conditions

The bacterial strains and plasmids used in this study are listed in **Table 1**. *E. coli* XL1 was used for cloning and plasmid propagation purposes and was cultured on solid or liquid Luria-Bertani medium. *M. bovis* BCG Pasteur 1173P2 (BCGwt) and *M. bovis* BCG_2177::Tn*gfp* (BCG-Tn) strains were routinely grown in 7H9 Middlebrook medium supplemented with 10% ADC and 0.05% Tween 80 or 7H10 Middlebrook medium supplemented with 10% OADC. When required, kanamycin (20 μg/ml), hygromycin (50 mg/ml), and sucrose (2%) were added to the growth medium. To study the involvement of the BCG_2177c (specific-targeted loci and TetR-like gene) in mycobacterial cholesterol catabolism, BCGwt and BCG-Tn were grown in Dubos broth (Difco^TM^) supplemented with 10% ADC to reach an optical density of 0.5 at 600 nm (OD_600_). This culture was used (1:10 dilution) to inoculate (i) Dubos broth base (without glycerol; BD Difco) containing 0.5% bovine serum albumin (BSA; fraction V, Sigma) and supplemented with 0.01% cholesterol [a 5% cholesterol stock was obtained by dissolving the sterol in 1:1 (v/v) tyloxapol-ethanol mixture, followed by heating for 5 min at 80°C], or (ii) Dubos medium with 10% ADC enrichment. Cultures were grown with agitation at 200 rpm at 37°C, and growth was monitored by measuring the OD_600_ and colony forming units per ml (CFU/ml).

**Table 1:** Characteristics of bacterial strains and plasmids used in this study.

Strains	Characteristics	Reference/Source
*E. coli* XL1	recA1 laclq lacZΔM15T*v*10	Sambrook et al., 1989
*M. bovis* BCG Pasteur	Vaccine strain 1173P2	Institute Pasteur Collection
**Plasmids**		
pBlueScript SK	Polylinker in lacZ gene and Amp^R^	Stratagene
pPR27	Ts, ori myco, *sac*B gene	Pelicic et al., 1997
pKEN-mut2	Vector containing *gfp* gene	Cormack et al., 1996
pPEP1	pBlue Script SK+*gfp* gene whithout promoter	This work
pPEP3	pBlue Script+ derivated of Tn*5367*	This work
pEZ120	pEP1 + IR	This work
pEZ121	pEZ120 + frag SpeI of pEP3	This work
pEZ123	pPR27 XbaI + Tn*gfp*	This work

### Introduction of Tn*gfp* in *M. bovis* BCG and Construction of Mutant Library

To produce insertion mutants, pEZ123 was introduced into *M. bovis* BCG by electroporation (Parish and Stoker, 1998). Cells were grown in plates with kanamycin at 32°C. Then single colonies were grown in broth at 32°C to allow transposition and subsequently plated on kanamycin- (selecting transposon presence) and sucrose-containing medium plates at 39°C, which are conditions not permissive for plasmid replication (Pelicic et al., 1997), allowing us to obtain the transposition mutants.

### Analysis of Mutants

The colonies that survived at 39°C on plates with sucrose and kanamycin were analyzed in order to detect transposition events. Mutant DNA digested with *BamH*I enzyme generates an internal fragment of the transposon, equal for all mutants, and two variable fragments depending on the region where the transposon was inserted (**Figure 2A**). Some colonies of *M. bovis* BCG were analyzed by Southern-blot (**Figure 2B**) and Ligation-Mediated PCR (LMPCR; Prod’hom et al., 1998).

All DNA manipulations, restriction enzyme digestions, agarose gel electrophoresis and Southern hybridizations were performed following standard procedures (Sambrook et al., 1989).

### Analysis of the Mutant Library by Flow Cytometry

The mutant libraries of *M. bovis* BCG were grown up at exponential phase, harvested by centrifugation, washed with PBS and resuspended in the same buffer. The bacterial suspensions were analyzed by flow cytometry (Coulter Epic “Elite”). Approximately 10.000 individual events were excited at 488 nm prior analysis.

### Isolation of *M. bovis* BCG Mutants Using Fluorescence Activated Cell Sorter (FACS)

To identify mycobacterial genes putatively involved in dormancy, the different pools of colonies from the *M. bovis* BCG::Tn*gfp* library were grown up to stationary phase, then flow cytometry was used to sort out cells by fluorescence emission. Bacteria from the *M. bovis* BCG::Tn*gfp* library were analyzed and sorted. After growth in 7H9 medium to stationary phase, BCG::Tn*gfp* cells were harvested and washed two times in 1X PBS. After the final wash bacteria pellets were resuspended in PBS (approx. 10^6^ bacteria/ml) and analyzed by FACS (Coulter Epics Elite sorter. Hielary Fl, USA). To do so, the pellet of bacteria was excited with 488-nm laser and 530 nm was applied for detection. Up to 3000 cells per second of sorting was used. One cycle of flow cytometric cell sorting was performed to select bacteria showing high levels of fluorescence. Bacteria exhibiting high levels of fluorescence were collected and were plated directly on 7H10 solid medium containing kanamycin to recover individual clones.

Different colonies were re-isolated and analyzed by LMPCR (Prod’hom et al., 1998) to identify transposon insertion sites.

### Location of Tn*gfp* Insertion Sites

In order to study Tn*gfp* insertion sites LMPCR was used according to the protocol described by Prod’hom et al. (1998). This technique allowed the amplification of both ends of the transposon. Briefly, the DNA was digested with the *Sal*I enzyme, the fragments of DNA were ligated with an adapter Sal, containing Salgd and Salpt sequences (**Table 2A**), and the resulting template was then digested with SalI. The PCR was performed using IS2 and gfp1 primers (specific for Tn*gfp* and directed outward) and the common linker primer Salgd (**Table 2A**). DNA was denatured by incubating the mixture at 95°C for 5 min. Amplification was achieved using 35 cycles of PCR (95°C for 30 s, specific annealing temperature for 30 s and 72°C for 90 s), followed by a final extension at 72°C for 10 m. Amplified products were separated by standard horizontal gel electrophoresis in a 1.5% agarose gel in TBE buffer (90 mM Tris, 90 mM boric acid, and 2 mM EDTA) and were stained using ethidium bromide. PCR products were purified using the GFX^TM^ PCR DNA and Gel Band Purification kit (Amersham Pharmacia Biotech Inc.) followed by ExoSAP-IT^®^ PCR product clean up (Affymetrix).

**Table 2:** Oligonucleotides used in this study.

**(A)** Oligonucleotides used for the construction and study of Tn*gfp* mutant library.			

**Target**	**Oligonucleotide Sequence (5′ – 3′)**		**Reference**

Insertion site	Salgd: TAGCTTATTCCTCAAGGCACGAGC		Prod’hom et al., 1998
	Salpt: TCGAGCTCGTGC	
Tn*gfp*	IS2: GAGGCGGCAGAAAGTCGTCAGGTCAG		This work
	gfp1: TCCTTCTTAAATCTAGGGCTGCAG	
Inverted Repeat (IR)	IR1: AAGCTTTCTAGAGGCTCTTCGCACTTGACGGTGTAGAGACGATCAGCTG CTTTCGCGCTGAAGCTT		This work
	IR2: TGCAGATCACAGCGGAAAGCAGCTGATCGTCTCTACACCGTCAAGTGCGAAGAGCC TCTAGAAGCTT	

**(B)** Oligonucleotides used for Real Time quantitative PCR (RT-qPCR) experiments^∗^.			

**Target**	**Oligonucleotide Sequence (5′ – 3′)**	**T°C ann.**	**Reference**

**16S ribosomal RNA**
*rrs* (16S rDNA)	16S TB-F: ATGACGGCCTTCGGGTTGTA	66°C	Cubero et al., 2013
	16S TB-R: CGTATTACCGCGGCTGCTGGCAC		
**Genes**
*tgs*1 (Rv3130c)	*tgs*1-F: TTGCCACCCGCCTTCC	50°C	This work
	*tgs*1-R: TCGCCACGGTGACAACA		
*ltp*2 (Rv3540c)	Rv3540c-F: GTATCGGGCATTCAACGAAC	55°C	This work
	Rv3540c-R: GTGCGGATACGAAAACGAAT		
*yrb*E4A (Rv3501c)	Rv3501c-F: CTACGCGTTCTCGGTCTTTC	55°C	This work
	Rv3501c-R: AGTATCAATTCGCGCAGTCC		
*mur*E (Rv2158c)	Rv2158c-F: CGCCTTCACCAATCTCTCC	55°C	This work
	Rv2158c-R: AGTCCGGATCGAACAATGAC		
**Small RNAS**
MTS2823	MTS2823-F: GAGAAGGTTCGGTCTCCCGAC	58°C	This work
	MTS2823-R: TTACGCAGACCCGCAACACT		
MTS0997	MTS0997-F: AAGCAGGCCCGGTTAGTGA	58°C	This work
	MTS0997-R: CACCGGTACACATGGGCAGA		
*rnp*B	rnpB-F: TTCACAGAGCAGGGTGATTG	62°C	This work
	rnpB-R: CTCTTACCGCACCGTTTCAC		

*^∗^The sequences of *M. tuberculosis* H37Rv and *M. bovis* BCG Pasteur are identical in all the genes and genomic regions under study.*

The amplified products were sequenced with the corresponding oligonucleotides using CNIO^3^ service and further checked for homology at Tuberculist^4^, Bovilist^5^ and NCBI^6^ database BLAST analysis.

### Microscopy Analysis

Mycobacterial cells were recovered from Dubos medium containing either dextrose or cholesterol at days 7 and 30, and were concentrated by centrifugation. Twenty microliter of each sample were smeared on three different slides, which were stained by Ziehl–Neelsen and with either, Auramine-O (Tec-Lam SA) or Nile Red (Sigma-Aldrich), following a previously published protocol (Deb et al., 2009). Microscopy for the fluorescence-based experiments was performed with an ECLIPSE Ti-Nikon’s microscope. Images were acquired using a Nikon Intensilight C-HGFI digital camera. All magnifications were carried out with a 100X oil-immersion objective.

### Mycobacterial RNA Isolation

Total RNA was isolated from cultures at the exponential and stationary phases as previously described (Gonzalez-y-Merchand et al., 1997). Briefly, cultures were harvested by centrifugation and pellets were resuspended in guanidinium chloride buffer (6M guanidinium chloride, 0.1% Tween 80, 1mM 2-mercaptoethanol, 10 mM EDTA) in a proportion of 1 ml of buffer/100 ml of culture. Cells were lysed mechanically in a FastPrep (Thermo Scientific) with 150- to 200-μm glass beads (Sigma-Aldrich) by performing four lysis cycles of 15 s each at high speed (6.5 m/s). Nucleic acids were purified with phenol-chloroform-isoamylic alcohol (25:24:1), and RNA was differentially precipitated with 0.4 volume of absolute ethanol (added drop by drop).

Finally, RNA was washed three times with Trizol reagent (Invitrogen). RNA integrity was analyzed with a bioanalyzer (Agilent technologies) and quantified by spectrophotometry with a NanoDrop ND-1000 (Thermo Scientific).

### qRT-PCR of Selected Genes and Non-coding RNAs

Transcription levels of several selected targets were measured by using real-time quantitative RT-PCR. Quantification was carried out with specific primers (**Table 2B**) and Light Cycler FastStart DNA SYBR green (Roche). Samples were subjected to 1 cycle at 95°C for 60 s (to activate the enzyme) followed by 40 cycles of amplification (denaturation at 95°C for 0 s, specific annealing temperature for 5 s, and extension at 72°C for 10 s) with a final extension at 72°C for 10 min. A melting curve analysis was performed to determine the specificity of the amplified product.

The standard curve was obtained for each pair of primers by using 10-fold dilutions of known amounts of *M. tuberculosis* H37Rv chromosomal DNA (nanograms of DNA per microliter). Threshold cycle values of each transcript quantified were interpolated to standard curve to measure the level of transcription. At least three amplifications per target were performed. Normalization of the data was performed by using 16S rRNA levels.

## Results

### Construction of the Tn*gfp* Delivery Plasmid pEZ123

**FIGURE 1:**
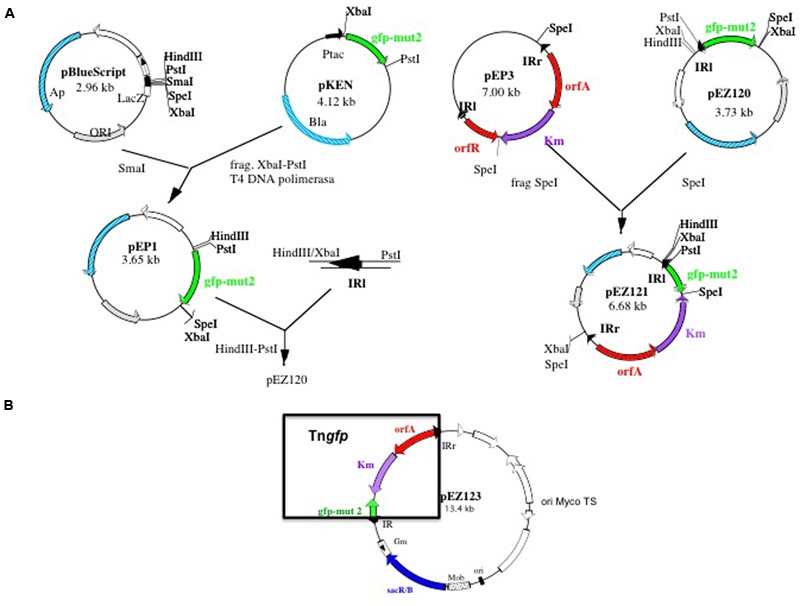
**Schematic diagram showing the construction of Tn*gfp* delivering system pEZ123. (A)** Construction of pEZ121 containing Tn*5367* derivative with *gfp* reporter gene (Tn*gfp*). **(B)** Map of the plasmid pEZ123 containing Tn*gfp*. This plasmid was used for the construction of the libraries of mutants of *Mycobacterium bovis* BCG. Delivering system is based on a thermosensitive and sensitive to sucrose mycobacterial plasmid. Construction is described in material and methods.

In order to obtain a library of mutants by transposition, containing a *gfp* gene, acting as reporter gene, an artificial transposon containing two IR of IS*1096* at both ends, was constructed as described in the Section “Materials and Methods” (**Figure 1A**). The transposon, named Tn*gfp* (**Figure 1B**), contains the *orfA* gene from Tn*5397* (Mcadam et al., 1995), a kanamycin-resistance gene and a promoterless copy of the gene encoding a mutated version of GFP (Cormack et al., 1996). This form of GFP exhibits greater fluorescence and solubility compared to the wild- type protein. Tn*gfp* was cloned into the plasmid pPR27 (Pelicic et al., 1997), an *Escherichia coli*-*Mycobacterium* shuttle plasmid, which is thermosensitive and able to replicate in mycobacteria, contains the gentamicin resistance gene and contains the *sacB* gene as a counter selectable marker. The resulting deliverable vector containing Tn*gfp* was named pEZ 123 (**Figure 1B**).

### Tn*gfp* Mutagenesis Library in *M. bovis* BCG

**FIGURE 2:**
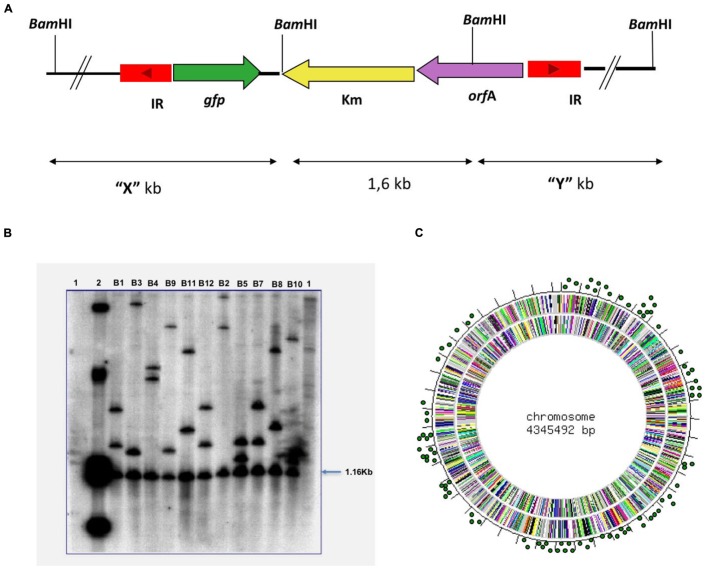
**Results of the Tn*gfp* transposition.** *M. bovis* BCG mutants analyzed by enzymatic digestion and then hybridized with a transposon probe. **(A)** Mutant DNA digested with BamHI enzyme, generates an internal fragment of the transposon (1.6 Kb), equal for all mutants, and two variable fragments depending on the region in which the transposon was inserted. **(B)** Southern-Blot analysis of 11 *M. bovis* BCG mutants. The DNA of colonies that had grown at 39°C with sucrose was digested with BamHI and hybridize with the Tn*gfp* like probe. Lane 1, DNA from *M. bovis* BCG; Lane 2, Plasmid pEZ123; Lanes B1–B12 different mutants of *M. bovis* BCG. **(C)** Distribution of Tn*gfp* through the *M. bovis* BCG genome. The obtained locations of the transposon were plotted in *M. bovis* BCG genome.

To determine the functional activity of Tn*gfp*, pEZ123 (**Figure 1B**) was introduced in the slow growing non-pathogenic mycobacteria *M. bovis* BCG. After growing on kanamycin and sucrose containing medium at 39°C, we selected transposition mutants. A transposon mutagenesis library with Tn*gfp* in BCG was constructed. We prepared an ordered library consisting of independent 5664 kanamycin, sucrose and thermoresistant mutants. The library was assembled into 40 pools. To verify that the transposition library was representative, some *M. bovis* BCG mutants were analyzed by digestion and then hybridized with the transposon. Mutant DNA digested with BamHI enzyme, generates an internal fragment of the transposon, equal for all mutants, and two variable fragments depending on the region where the transposon was inserted (**Figure 2A**). Southern blot analysis of randomly selected colonies for 11 mutants showed that each of them contained a single transposon insertion that produced a unique restriction fragment pattern (**Figure 2B**). We also analyzed 150 mutants from the ordered library by LMPCR technique. By sequencing the transposon flanking regions, we identified 77 different points of transposon insertion (Supplementary Table S1). The obtained locations of the transposon were plotted in the *M. bovis* BCG genome showing a distribution through the entire chromosome length (**Figure 2C**). Sequencing of the insertion junctions of the 77 mutants revealed that 66 (85.7%) insertions were found within ORFs and 11 were intergenic regions (Supplementary Table S1).

### Flow Cytometry Analysis of the Tn*gfp M. bovis* BCG Libraries

To determine if the *M. bovis* BCG mutant library was useful to differentiate populations of fluorescent bacteria, the library was analyzed by flow cytometry. Only 3.65% of the *M. bovis* BCG mutants in the library showed detectable fluorescence levels (**Supplementary Figure S1A**).

**FIGURE 3:**
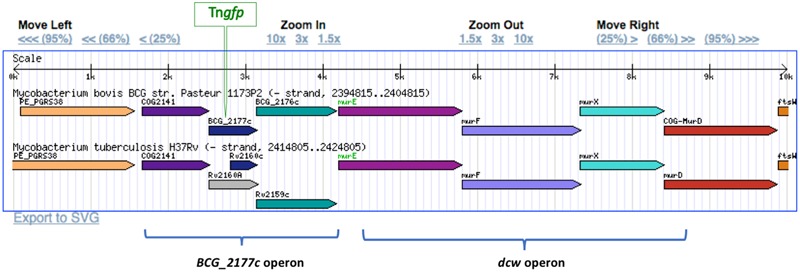
**Location of Tngfp in BCG_2177c gene in the *M. bovis* BCG genome.** BCG_2177c gene is located upstream from the division cell wall gene cluster (*dcw*). The insertion of Tngfp was in the same orientation that replication *dcw* genes. Gene members of the *dcw* operon as well as genes of the same operon that BCG_2177c were indicated.

Aiming to select transposition mutants putatively involved in dormancy, further analyses were performed to detect *M. bovis* BCG::Tn*gfp* cells exhibiting high levels of fluorescence at stationary phase by using FACS. Up to 23 colonies were re-isolated and analyzed by LMPCR to localize the transposon insertion site. From these, only mutants where the orientation of *gfp* and the neighbor gene was the same were selected. One of those mutants showed GFP expression level more than 20-fold compared to other mutants. GFP production was maximal at stationary phase (**Supplementary Figure S1B**). This mutant was identified as carrying the transposon inserted 56 nucleotides inside from start codon of the 2177c gene of *M. bovis* BCG, which is part of an operon located immediately upstream of the *dcw* operon (**Figure 3**), the last known to be involved in cell division and cell wall formation in *M. tuberculosis* (Munshi et al., 2013).

BCG_2177c has been reported to be homologous to Rv2160A of *M. tuberculosis*, identified as a member of the TetR-family of transcriptional regulators, which have been related to the regulation of genes involved in cholesterol metabolism (Casabon et al., 2013).

### Growth of BCG-Tn in Cholesterol

In order to determine the influence of cholesterol on the growth of (BCG-Tn), we cultured BCGwt and its mutant in a medium supplemented with cholesterol as a sole carbon source. Growth in cholesterol was compared to growth in dextrose (control medium). We found similar growth profiles measured by CFUs for those mycobacteria independent of the carbon source used (**Supplementary Figure S2B**). Some differences were found in the growth profiles measured by OD. For example, the exponential phase slopes of the wild type strain and its corresponding mutant were very similar in the presence of cholesterol (**Supplementary Figure S2A**) and both strains reached the stationary phase at the same time (day 4). In contrast, when both mycobacteria were grown in the presence of dextrose, BCG-Tn showed an exponential phase delay, compared with wild type, and it approximately reached only half of the cell density (in relation to wild type) at the end of stationary phase. The influence of cell aggregation in the OD results cannot be discarded.

**FIGURE 4:**
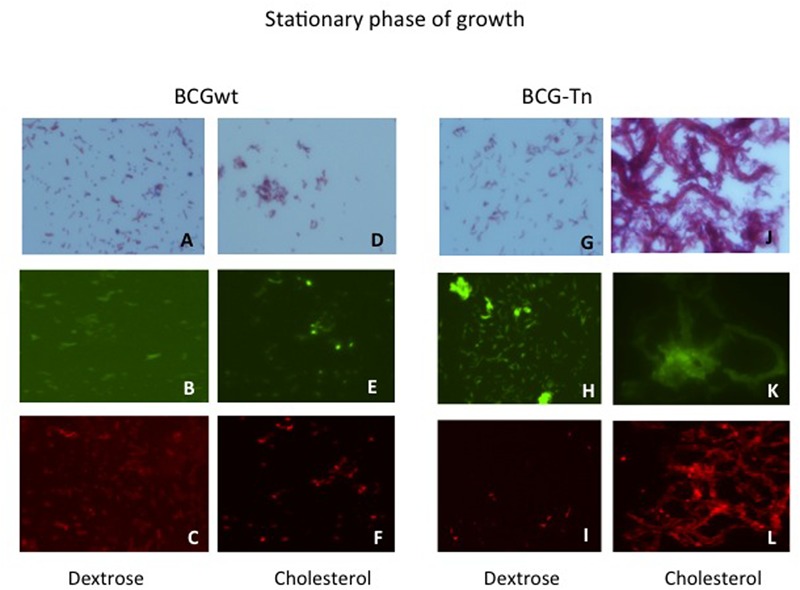
**Morphological changes of BGC wt and BCG-Tn during stationary phase in the presence of different carbon sources.** Morphological changes in stationary phase of mycobacterial cultures after growth in the presence of dextrose **(A–C)** and **(G–I)** or cholesterol **(D–F)** and **(J–L)** as carbon sources. BCGwt **(A–F)** or BCG-Tn **(G–L)** cells were harvested from the corresponding cultures and stained by Ziehl–Neelsen **(A,D,G,J)** or with Auramine **(B,E,H,K)** or Red Nile **(C,F,I,L)** the two last by fluorescent microscopy. Cord-like structures are observed in **(J–L)** panels, corresponding to stationary phase of the mutant growing in cholesterol. All magnifications were carried out with the 100X oil immersion objective.

The effect of cholesterol on the microscopic morphology of BCG wild type and BCG-Tn mutant was analyzed at exponential and stationary growth phases. Both strains were tested and their growth in dextrose and cholesterol were also compared (**Figure 4** and **Supplementary Figure S3**). When both strains were cultured in the presence of cholesterol, an accumulation in the lipid content was shown, by Nile Red stain, compared to the cultures in dextrose. During the exponential phase of growth, no differences were detected comparing wild-type and mutant by Ziehl–Neelsen, Auramine or Red Nile stains (**Supplementary Figure S3**). In contrast, strong cord formation was identified at the stationary phase when the mutant was grown in cholesterol (**Figures 4J–L**). In addition, cell aggregation was also found in the wild-type growing in cholesterol at stationary phase (**Figures 4D–F**). The aggregates or cords were not present in stationary phase when wild-type and mutant were grown in dextrose (**Figures 4A–C,G,H**).

### Gene Expression of *M. bovis* BCG-Tn in Cholesterol

To gain insights into the influence of the transposon on gene expression related to cholesterol metabolism, several genes were quantitatively analyzed from cultures recovered at exponential and stationary phases of growth. Genes related to cholesterol metabolism such as *ltp*2, a probable lipid transfer protein, and *yrb*E4A, a possible ABC transporter, both putatively involved in cholesterol intake, were studied. Apart from *tgs*1, a central gene in the lipid metabolism and *mur*E, the gene leading the *dcw* operon located downstream of the transposon insertion was also studied. Comparisons were made between BCGwt and BCG-Tn growing with and without cholesterol as a sole carbon source. Moreover, cultures were recovered at exponential and stationary phases of growth in each culture condition.

**FIGURE 5:**
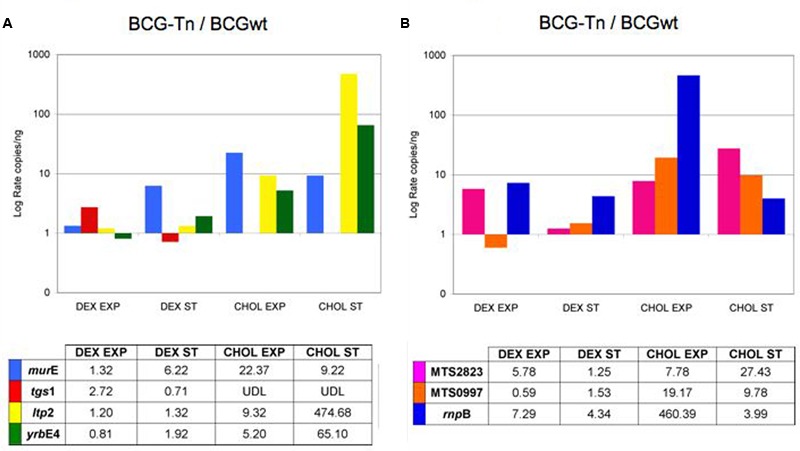
**Differential expression of selected genes and ncRNA in *M. bovis* BCG (BCGwt and BCG-Tn).** Level of expression of BCG wt versus BCG-Tn of **(A)** *murE, tgs*1, *ltp*2, and *yrb*E4A genes; and **(B)** MTS2823, MTS0997, and *rnp*B ncRNAs. Log rate of number of copies/ng of the mutant BCG-Tn and BCGwt were calculated in different conditions of culture (Dextrose: DEX; Cholesterol: CHOL) and growing phases (Exponential: EXP; Stationary: ST). The data were normalized with the expression of *rrs* gene coding for 16S-rRNA. UDL, under detection level. Complete set of data is showed in **Supplementary Figures S4, S5**.

**Figure 5** showed the ratio of gene expression comparing mutant versus wt bacteria. The level of expression of *mur*E was higher in the mutant, particularly when bacilli grew in cholesterol and during exponential phase. Genes related to cholesterol metabolism, namely *ltp*2 and *yrb*E4A, also showed higher expression in the mutant compared to the wt when bacteria grew with cholesterol, with a more marked increase observed during stationary phase (**Figure 5A**). These results could suggest a repressor activity of the *tet*R-like gene over those genes, being that activity enhanced in the presence of cholesterol. On the contrary, the level of expression of *tgs*1 was lower in dextrose and stationary phase in the mutant compared to the wt. This gene was undetectable in the mutant when the bacteria grew in cholesterol (**Figure 5A**).

Detection of non-coding RNAs was also performed (**Figure 5B**). With few exceptions, the three non-coding targets showed higher expression in the mutant compared to the wt, both in dextrose as well as in cholesterol. The non-coding *rnp*B, involved in tRNA maturation was 400-fold more expressed in the mutant during exponential phase in cholesterol (**Figure 5B**).

The complete set of qRT-PCR normalized data of the targeted genes and the targeted non-coding RNAs, are presented in **Supplementary Figures S4, S5**, respectively.

## Discussion

Since transposition was first demonstrated in fast growing mycobacteria with IS*6100* (Martin et al., 1990), other mycobacterial ISs, not presents in *M. tuberculosis*, have been demonstrated to be active in mycobacteria (Mcadam et al., 2000). Transposons derived from the *M. smegmatis* insertion sequence IS*1096* (Cirillo et al., 1991) were modified for its use in slow-growing mycobacteria by the insertion of a kanamycin resistance cassette showing to be active in *M. bovis* BCG and in several strains of *M. tuberculosis* and *M. paratuberculosis* (Mcadam et al., 1995; Bardarov et al., 1997; Pelicic et al., 1997; Harris et al., 1999). After those developments, transposon mutagenesis was available in mycobacteria allowing for the preparation of transposon-mutant libraries, in such a way that phenotypic screening on a large scale could be undertaken, including the study of genes required for pathogen adaptation to a host by using signature-tagged mutagenesis (Camacho et al., 1999; Cox et al., 1999).

The use of fusions with reporter genes might enable the identification of genes that were differently expressed under specific conditions (Machowski et al., 2000; Dandie et al., 2006). The *gfp* gene is a useful reporter for *in vivo* studies because no substrate is required for the detection of its fluorescent product (Valdivia and Falkow, 1998). Previous studies allowed the detection of promoters through detection of an increase expression of *gfp* in *M. tuberculosis* and *M. bovis* BCG (Triccas et al., 1999) or *M. marinum* (Barker et al., 1998) growing inside macrophages. In our work, intracellular levels of GFP were found to be 2–20 times higher than that found in previous *in vitro* cultures of those mycobacteria performed in the laboratory (data not shown).

In this work, we have developed a new artificial transposon suitable to generate expression libraries of the GFP fluorescent protein in mycobacteria. We have tested the transposition driven by Tn*gfp* as a rapid method to generate multiple insertions into the chromosome of *M. bovis* BCG, and we constructed a comprehensive bank of 5664 insertion mutants using a new transposon mutagenesis system. Moreover, analysis by flow cytometry provided high sensitivity allowing the detection of fluorescent bacteria, even with a single copy of *gfp* per genome, therefore it was useful for the screening of *M. bovis* BCG transposon mutant libraries under different conditions.

A wide range of different insertion sites were identified in *M. bovis* BCG (up to 77/150 clones). When, we analyzed the transposon insertion points of these mutants we observed that transposition occurred randomly (**Figure 2C** and Supplementary Table S1). No consensus sequence was identified as a target for this transposon. Remarkably, 85,7% of the insertions happened in coding regions. The suitability of the procedure to generate a representative library could be inferred taking into consideration that: (1) 91% of *M. tuberculosis* genome corresponds to open reading frames (Cole et al., 1998); and (2) when the transposon disrupts an essential gene such a mutant will not be obtained. In addition, genes interrupted by Tn*gfp* are distributed among the different functional categories in the BCG genome, similarly to the proportional distribution in the genome of *M. tuberculosis*^7^. Other studies of transposition *in vivo* (using IS*6110)* reported only 50% transposon insertion within coding regions in *M. tuberculosis* and in *M. bovis* strains (Mendiola et al., 1992; Otal et al., 2008; Alonso et al., 2013).

In searching for transposition events in loci putatively related to dormancy, the fluorescent level of *M. bovis* BCG::Tn*gfp* cells were studied in stationary phase and analyzed by flow cytometry. Using this procedure, a mutant targeting the gene BCG_2177c (named BCG-Tn) was detected. This transposon mutant showed approximately 20 times higher fluorescent levels at stationary phase compared to other mutants tested (data not shown).

BCG_2177c is homologous to *M. tuberculosis* Rv2160A, a gene identified in *M. tuberculosis* with similarities to the TetR-family of transcriptional regulators. TetR is a large family of transcriptional regulators known to be involved in many metabolic routes in bacteria (Ramos et al., 2005). Some members of this family were involved in the metabolism of cholesterol (Klepp et al., 2012). The genome of *M. tuberculosis* encodes for a high number of genes involved in the catabolism of cholesterol (Ouellet et al., 2011), a main carbon source of the tubercle bacilli during infection, including latent infection (Miner et al., 2009).

To gain insights into the role that BCG_2177c could play in *M. bovis* BCG, BCG-Tn mutant and BCG *wt* were studied in more detail during their growth in cholesterol. Growth curves of both bacteria in cholesterol or dextrose as main carbon sources showed an impaired growth in dextrose of the mutant compared to *wt* (**Supplementary Figure S2**). On the other hand, a particular morphological change was observed in the mutant during stationary phase in cholesterol. Under this last condition, the BCG-Tn bacilli showed strong cord formation (**Figures 4J–L**), which was not observed in the mutant or in the *wt* in any other condition. These findings suggest changes in the cell envelope of the bacteria possibly related to the targeted gene.

Notable was the increased level of the nc RNA, namely rnpB, in the mutant, during exponential phase growth in cholesterol (**Figure 5**). This non-coding RNA is a component of the RNase P and participates in the maturation of tRNAs (Herrmann et al., 2014). The involvement of tRNAs in changes at the cell wall level (Francklyn and Minajigi, 2010) suggests that a modification of such cell structures during the adaptation of the mutant to growth in cholesterol could putatively be related with the phenotypic changes of the mutant in stationary phase detected by acid-fast and fluorescent staining (**Figures 4J–L**). Interestingly, this ncRNA seems to be essential for *M. tuberculosis* when the bacillus grows in cholesterol (Griffin et al., 2011).

To further analyze the influence of cholesterol in more detail, the transcriptomic patterns of selected genomic regions known to have some role in the infection of *M. tuberculosis* (Arnvig and Young, 2009; Miner et al., 2009) were studied. This was carried out in all four culture conditions. Results showed that genes related to cholesterol metabolism (*ltp*2 and *yrb*E4A) have roughly similar expression levels between mutant and *wt* in dextrose, but their levels of expression increased in the mutant during growth in cholesterol, mainly at stationary phase (**Figure 5A**). These results suggested a putative repressor activity of BCG_2177c on those genes in the presence of cholesterol. It has been shown that *yrb*E4 expression is regulated by a TetR-family member (KstR) in *M. tuberculosis* (Ouellet et al., 2011). The expression level of the *yrb*E4 is not affected when *M. tuberculosis* is cultured in dextrose^8^; unfortunately, this effect is unknown in *M. tuberculosis* growing in cholesterol. Our results suggested that BCG_2177c could play a similar role in *M. bovis* BCG, and that influence appeared to be enhanced during growth in cholesterol.

The participation of the gene *mur*E in the synthesis of the peptide-glycan, suggested a higher level of its expression when bacteria grows faster. As expected, according to **Supplementary Figure S2**, a higher level of expression of this gene was detected in cholesterol compared to dextrose in both strains. Compared to BCG *wt*, the level of expression detected for *mur*E in the mutant BCG-Tn was high in all conditions, except dextrose at exponential phase (**Figure 5A**), a condition that causes an impaired growth of the mutant (**Supplementary Figure S2**). Whether this result was a direct consequence of the transposition event or not requires further studies.

An unexpected result was found for the gene *tgs*1, a main gene involved in dormancy that participates in the synthesis of triacylglycerol in *M. tuberculosis*. Comparing mutant and *wt*, this gene was similarly expressed in both strains during growth in dextrose, although it showed a slight level of increase in exponential phase in the mutant. However, its expression was undetectable in the mutant when cholesterol was used as a carbon source (**Figure 5**). Our results showed that the expression of *tgs*1 ceased when BCG_2177c was truncated after transposition, thus suggesting a direct or indirect participation of the BCG_2177c gene in the activation of *tgs*1 expression in *M. bovis* BCG during growth in cholesterol.

Finally, the expression of two other nc-RNAs was also analyzed in the mutant BCG-Tn and BCG *wt* under the four conditions studied (**Figure 5B**). The data showed a higher level of expression of the nc-RNAs in the mutant, when bacteria grew in cholesterol, particularly at stationary growth phase. This result was also found when *M. tuberculosis* was grown in long chain-fatty acids as a sole carbon source (Rodriguez et al., 2014) thus suggesting a role of these two nc-RNAs during latent infection, a situation in which lipids, including cholesterol, seem to play an essential role in *M. tuberculosis*. Our results showed that BCG_2177c could play some role during a similar process in *M. bovis* BCG.

As described in this work, the transposon mutagenesis system has showed to be an excellent procedure in the study of gene function, even in fastidious bacteria such as mycobacteria. Our transposon Tn*gfp* was shown to be active also in other mycobacteria, from the main pathogen *M. tuberculosis* to the saprophyte *M. smegmatis*, therefore this methodology could be putatively applied to the study of gene function in other members of the genus. Moreover, by application of the Tn-seq technique, this transposon system represents a valuable tool for a wider study of gene function in the genus.

Finally, limitations of the system exist, such as the lack of detection of low-level promoters or the lost of strong promoters due to deleterious effect of GFP accumulation. Likewise, the involvement of other regulators that may indirectly influence the changes detected cannot be discarded.

In summary, to the best of our knowledge, this work showed for the first time the usefulness of a transposon mutant library prepared with the promoterless gfp gene. We have showed that the novel transposon mutagenesis system developed in this study is a useful tool to analyze functional activity of targeted genes in *M. bovis* BCG. While further characterization is still required, our work also proposes the direct or indirect involvement of BCG_2177c gene in cholesterol metabolism of this vaccine strain, suggesting a role of this putative TetR gene during the survival of the strain in the human host, a cholesterol-enriched environment.

## Author Contributions

EP-H constructed the transposon, IO prepared and studied the *M. bovis* library and selecting the mutant. LG-M, JG-M, MM, and MG studied the mutant in cholesterol. IO, JG-M, MM, and MG wrote the manuscript. LG-M, EP-H, and CM contributed to the final draft and revision. All authors read and approved the manuscript.

## Conflict of Interest Statement

The authors declare that the research was conducted in the absence of any commercial or financial relationships that could be construed as a potential conflict of interest.

The reviewer JM and handling Editor declared their shared affiliation and the handling Editor states that the process nevertheless met the standards of a fair and objective review.

## Footnotes

1 http://www.who.org

2 ftp://ftp.ncbi.nlm.nih.gov

3 https://www.cnio.es/

4 http://genolist.pasteur.fr/TubercuList

5 http://genolist.pasteur.fr/BoviList

6 http://www.ncbi.nlm.nih.gov/

7 http://genolist.pasteur.fr/TubercuList

8 http://www.tbdb.org

## Supplementary Material

FIGURE S1 **GFP expression of the *M. bovis* BCG library and *M. bovis* BCG-Tn. (A)** Flow cytometry analysis of the library of *M. bovis* BCG mutants at logarithmic phase. A total of 3.65% of the mutants showed detectable fluorescence level. **(B)** Histograms obtained by flow cytometry at four different points of the growth curve of the mutant *M. bovis* BCG-Tn. The expression level of gfp at stationary phase (days 32 and 64 of growth) was much higher than at logarithmic phase (days 2 and 3).

FIGURE S2 ***Mycobacterium bovis* BCGwt and *M. bovis* BCG-Tn growth curve in the presence of dextrose (DEX) or cholesterol (CHOL) as carbon sources. (A)** Optical density at 600 nm; **(B)** Colony forming units.

FIGURE S3 **Morphological changes of BGC wt and BCG-Tn during exponential phase in the presence of different carbon sources.** Morphological changes in exponential phase of mycobacterial cultures after growth in the presence of dextrose **(A–C)** and **(G–I)** or colesterol **(D–F)** and **(J–L)** as carbon sources. BCGwt **(A–F)** or BCG-Tn **(G–L)** cells were harvested from the corresponding culture and stained by Ziehl–Neelsen **(A,D,G,J)** or with Auramine **(B,E,H,K)** or Red Nile **(C,F,I,L)** the two last by fluorescent microscopy.

FIGURE S4 **Levels of expression of genes in *M. bovis* BCGwt and *M. bovis* BCG-Tn normalized with 16S rRNA.** Levels of expression in the presence of dextrose (DEX) or cholesterol (CHOL) during exponential (EXP) and stationary (ST) phases of growth. Mean values of at least three amplifications per target and corresponding error bars are indicated.

FIGURE S5 **Levels of expression of ncRNAs in *M. bovis* BCGwt and *M. bovis* BCG-Tn normalized with 16S rRNA.** Levels of expression in the presence of dextrose (DEX) or cholesterol (CHOL) during exponential (EXP) and stationary (ST) phases of growth. Mean values of at least three amplifications per target and corresponding error bars are indicated.
