# Supplementary material for: Detection of a Putative TetR-Like Gene Related to Mycobacterium bovis BCG Growth in Cholesterol Using a gfp-Transposon Mutagenesis System
Source: Front Microbiol. 2017 Mar 6;8:315. doi: 10.3389/fmicb.2017.00315 (PMC5337628; doi:10.3389/fmicb.2017.00315)
Supplement: Supplementary file 1 [file Table_1.DOC]

**TABLE S1.** Genes disrupted by Tn*gfp* insertion in the *M. bovis* BCG genome and their corresponding orthologue in the *M. tuberculosis* H37Rv.

| **BCG-Pasteur** | **H37Rv** |
| --- | --- |
| 0102 | 0071 |
| 0111c | 0078A |
| 0134 | 0101(*nrp*) |
| 0145 | 0112 |
| 0162 | 0128 |
| 0196c | 0160c |
| 0260c | 0223 |
| 0312 | 0274 |
| 0380 | 0341 *(ini*B) |
| 0425 | 0387 |
| 0441c | 0403c (*mmpS*1) |
| 0442 | 0404 (*fadD*30)* |
| 0443 | 0405 (*pks*6) |
| 0489 | 450c (*mmpL*4) |
| 0493 | 0454 |
| 0550 | 0507 (*mmpL*2) |
| 0643c | 0597c |
| 0805-0806c | 0754-0755c |
| 0939c-0940 | 0887c-0888 |
| 0940 | 0888* |
| 0944 | 0892 |
| 0983c | 0931c (*pknD*) |
| 1042 | 0987 |
| 1043-1044c | 0988-0989c (*grc*C2) |
| 1204c | 1142c (*ech*A10) |
| 1377c | 1316c (*ogt*) |
| 1418c-1419c | 1356c-1357c |
| 1553 | 1490 |
| 1564 | 1500 |
| 1565 | 1501 |
| 1567c | 1503c |
| 1566-1567c | 1502-1503c |
| 1574c | 1522c (*mmpL*12) |
| 1584c-1585 | 1532c-1533 |
| 1821 | 1789 (PPE26) |
| 1844 | 1810 |
| 2025c | 2008c |
| 2086c | 2067c |
| 2162c-2163c | 2145c (*wag*31)-2146c |
| 2177c | 2160A/2160c |
| 2306 | 2290 (*lppO*) |
| 2359c-2360c | 2337c-2338c (*moeW*) |
| 2360c | 2338c |
| 2361 | 2339 (*mmpL*9) |
| 2442c | 2425c |
| 2714c-2715 | 2701c- 2702 (*suh*B-*ppg*K) |
| 2744 | 2731 |
| 2748c | 2735c |
| 2951-2952 | 2929-2930 (*fadD*26) |
| 2952 | 2930 (*fadD*26) |
| 2957 | 2935* |
| 2963 | 2941 (*fadD*28) |
| 2965 | 2943 |
| 2971 | 2950c (*fadD*29) |
| 3119c | 3094c |
| 3134 | 3109 (*moa*A1) |
| 3138 | 3113 |
| 3154 | 3131 |
| 3182 | 3159c (PPE53) |
| 3239 | 3212 |
| 3252 | 3224B |
| 3359c | 3294c |
| 3362 | 3297(*nei*) |
| 3409c-3410 | 3339c-3340 (*icd*1-*met*C) |
| 3449c | 3377c |
| 3494c | 3424c |
| 3659 | 3594 |
| 3787 | 3727 |
| 3865c | 3803c (*fbp*D) |
| 3886c | 3823c (*mmp*L8) |
| 3888c | 3825c (*pks*2) |
| 3889 | 3826 (*fad*D23) |
| 3890c | 3827c |
| 3960c | 3903c |

* Two different insertion points.
