# Supplementary figures and images for: Detection of a Putative TetR-Like Gene Related to Mycobacterium bovis BCG Growth in Cholesterol Using a gfp-Transposon Mutagenesis System
Source: Front Microbiol. 2017 Mar 6;8:315. doi: 10.3389/fmicb.2017.00315 (PMC5337628; doi:10.3389/fmicb.2017.00315)

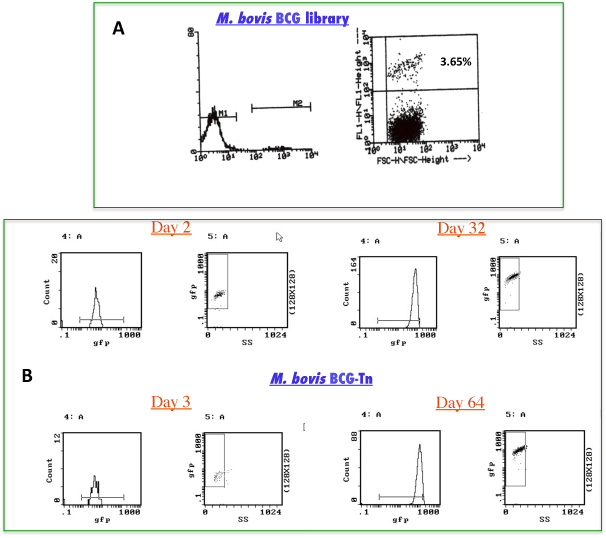

Supplement: FIGURE S1 — GFP expression of the M. bovis BCG library and M. bovis BCG-Tn. (A) Flow cytometry analysis of the library of M. bovis BCG mutants at logarithmic phase. A total of 3.65% of the mutants showed detectable fluorescence level. (B) Histograms obtained by flow cytometry at four different points of the growth curve of the mutant M. bovis BCG-Tn. The expression level of gfp at stationary phase (days 32 and 64 of growth) was much higher than at logarithmic phase (days 2 and 3). [file Image_1.JPEG]

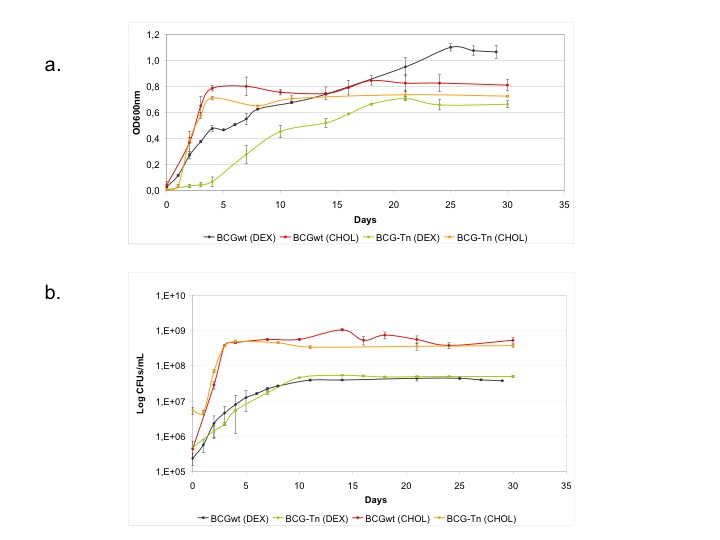

Supplement: FIGURE S2 — Mycobacterium bovis BCGwt and M. bovis BCG-Tn growth curve in the presence of dextrose (DEX) or cholesterol (CHOL) as carbon sources. (A) Optical density at 600 nm; (B) Colony forming units. [file Image_2.JPEG]

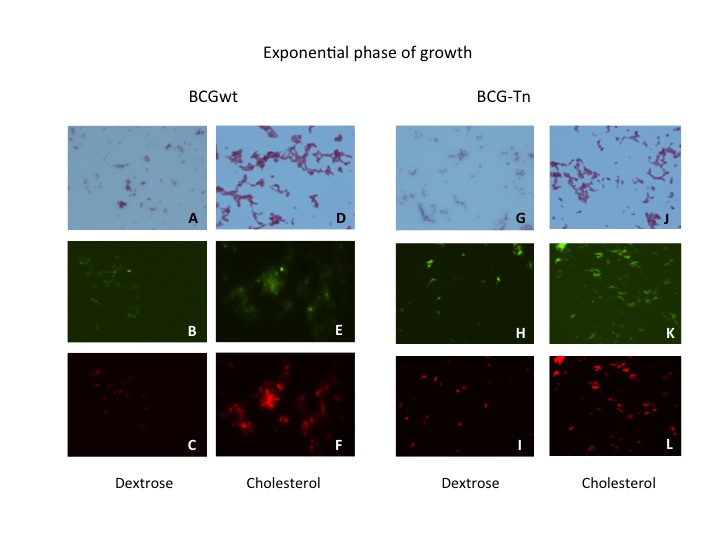

Supplement: FIGURE S3 — Morphological changes of BGC wt and BCG-Tn during exponential phase in the presence of different carbon sources. Morphological changes in exponential phase of mycobacterial cultures after growth in the presence of dextrose (A–C) and (G–I) or colesterol (D–F) and (J–L) as carbon sources. BCGwt (A–F) or BCG-Tn (G–L) cells were harvested from the corresponding culture and stained by Ziehl–Neelsen (A,D,G,J) or with Auramine (B,E,H,K) or Red Nile (C,F,I,L) the two last by fluorescent microscopy. [file Image_3.JPEG]

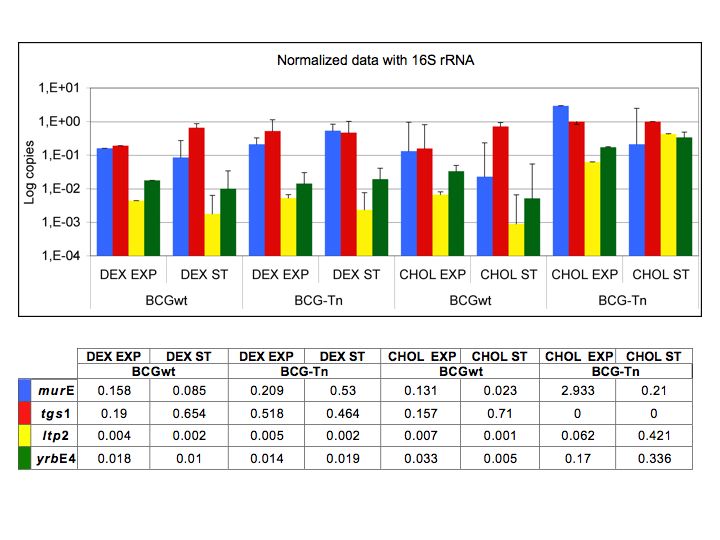

Supplement: FIGURE S4 — Levels of expression of genes in M. bovis BCGwt and M. bovis BCG-Tn normalized with 16S rRNA. Levels of expression in the presence of dextrose (DEX) or cholesterol (CHOL) during exponential (EXP) and stationary (ST) phases of growth. Mean values of at least three amplifications per target and corresponding error bars are indicated. [file Image_4.JPEG]

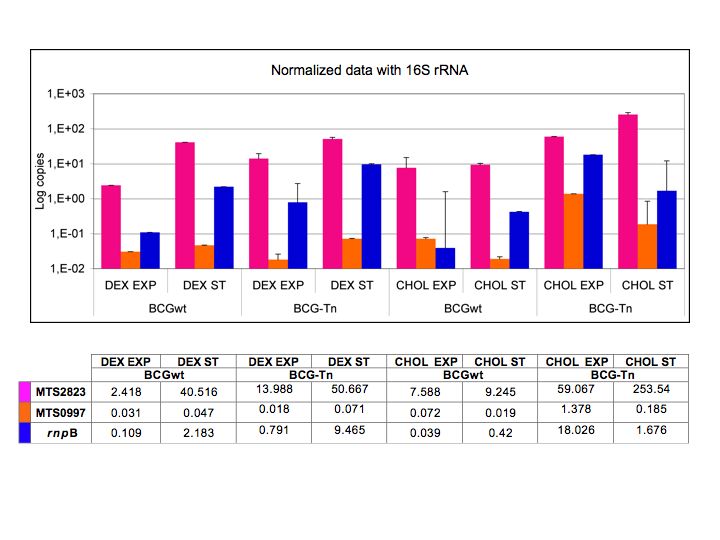

Supplement: FIGURE S5 — Levels of expression of ncRNAs in M. bovis BCGwt and M. bovis BCG-Tn normalized with 16S rRNA. Levels of expression in the presence of dextrose (DEX) or cholesterol (CHOL) during exponential (EXP) and stationary (ST) phases of growth. Mean values of at least three amplifications per target and corresponding error bars are indicated. [file Image_5.JPEG]
